# NGSQC: cross-platform quality analysis pipeline for deep sequencing data

**DOI:** 10.1186/1471-2164-11-S4-S7

**Published:** 2010-12-02

**Authors:** Manhong Dai, Robert C Thompson, Christopher Maher, Rafael Contreras-Galindo, Mark H Kaplan, David M Markovitz, Gil Omenn, Fan Meng

**Affiliations:** 1 Department of Psychiatry and Molecular and Behavioral Neuroscience Institute, University of Michigan, Ann Arbor, MI 48109, USA; 2 Michigan Center for Translational Pathology, Department of Pathology, University of Michigan, Ann Arbor, MI 48109, USA; 3 Division of Infectious Diseases, Department of Internal Medicine, University of Michigan, Ann Arbor, MI 48109, USA; 4 Center for Computational Medicine and Biology, University of Michigan, Ann Arbor, MI 48109, USA; 5 National Center for Integrative Biomedical Informatics, University of Michigan, Ann Arbor, MI 48109, USA

## Abstract

**Background:**

While the accuracy and precision of deep sequencing data is significantly better than those obtained by the earlier generation of hybridization-based high throughput technologies, the digital nature of deep sequencing output often leads to unwarranted confidence in their reliability.

**Results:**

The NGSQC (Next Generation Sequencing Quality Control) pipeline provides a set of novel quality control measures for quickly detecting a wide variety of quality issues in deep sequencing data derived from two dimensional surfaces, regardless of the assay technology used. It also enables researchers to determine whether sequencing data related to their most interesting biological discoveries are caused by sequencing quality issues.

**Conclusions:**

Next generation sequencing platforms have their own share of quality issues and there can be significant lab-to-lab, batch-to-batch and even within chip/slide variations. NGSQC can help to ensure that biological conclusions, in particular those based on relatively rare sequence alterations, are not caused by low quality sequencing.

## Background

Rapid advances in our understanding about the molecular mechanisms underlying different pathological processes in the last decade or so have been largely catalyzed by various hybridization-based high throughput methods, which enable biomedical researchers to explore the potential contribution from almost all known genes and genomic structures. However, most hybridization-based approaches are still constrained by our current understanding of genome and transcriptome, as we can only include probes for known targets. This can be a significant limitation for areas about which we have relatively little knowledge, such as alternative splicing, transcription factor binding and genome methylation. In addition, the limited dynamic range and the noisiness of hybridization signals also constrain their usefulness in many studies.

In contrast, the next generation sequencing technologies provide the possibility of comprehensive sampling of the genome/transcriptome in the absence of knowledge about their potential variation, as sequence readouts provide both structure variation and quantity information without relying on pre-designed probes. In addition, the final sequencing readouts are digital in nature and they should cover a much broader dynamic range as well as provide more accurate quantitative values. Consequently, deep sequencing is quickly becoming the preferred method for many genome and transcriptome studies. It can be expected that the rapid advancement of various ultra-high throughput technologies will likely make the sequencing-based approaches even more popular for a wide variety of studies including gene expression, transcription factor binding, genomic structural change, genome methylation and even *de novo* genome assemblies.

However, due to the digital nature of sequencing output, most biomedical researchers tend to believe that although sequencing data will contain platform-specific base calling errors and possibly small insertions/deletions, it is unlikely that such sequencing errors will lead to qualitatively incorrect conclusions. In addition, since deep sequencing platform vendors already provide quality scores and filters for sequencing output, most users would think that there should be no major quality issues in sequencing if the vendor data analysis pipelines are set up properly. This attitude is evident in the fact that although three major deep sequencing platforms from Illumina, Applied Biosystems and Roche have been in widespread use for at least two years, there are only two published third party quality control (QC) packages, TileQC[1] and PIQA[2] , both of them targeted the Illumina platform.

Regardless of the specific technologies used, sequencing assays are very complicated processes and the final output can be influenced by many factors including fluidics, optics, assay setup and sequencing library preparation. The complexity of the sequencing process also means that there will be lab-to-lab and batch-to-batch variations. In addition, identifying quality problems that have significant impact on biological conclusions requires in-depth understanding of the biological problem and considerable efforts for linking sequence reads to assay quality issues. Consequently, it is unrealistic to expect typical assay vendors to proactively devote significant resources to identify all major problems in their assays under different situations. In fact, this is what we have learned from the use of microarray platforms: high throughput assay vendors are mainly focused on assay/hardware development. Data analysis solutions provided by vendors are workable but can often be significantly improved by third party users and developers [3-8].

In our own deep sequencing data analysis projects, we came across a number of issues, such as uneven base percentage across assay surface and large scale low quality patterns that neither QC solutions from sequencing platform vendors or third party quality (i.e., TileQC and PIQA) can detect. Besides lacking some important QC measures, existing deep sequencing QC solutions emphasize quality analysis at the individual sequence or the individual sequencing data capture tile/panel level. It is difficult to identify quality change trends or patterns across the whole sample, which usually consists of 100 to over 2000 tiles/panels. In addition, existing solutions do not provide an easy way to identify biological consequences of deep sequencing quality issues. It is a very time-consuming task for a typical biomedical researcher to verify that sequence reads related to an interesting biological conclusion are not the result of quality issues in the sequencing assay. Moreover, available third party deep sequencing QC solutions are designed for the Illumina platform only. There is no third party QC package that can deal with QC needs for the widely used Applied Biosystems SOLiD platform or for future generations of deep sequencing platforms.

Our NGSQC (Next Generation Sequencing Quality Control) pipeline is designed to address the above shortcomings in deep sequencing quality analysis. NGSQC provides comprehensive quality control measures at sequence, tile/panel and sample levels. It can be quickly adapted for new sequencing platforms that perform sequencing on two dimensional surfaces. NGSQC also allows biomedical researchers to quickly identify biologically relevant quality issues in their deep sequencing data and to make sure that sequences related to specific biological conclusions are not the result of sequencing quality problems.

## Implementation

### New quality control measures

All sequencing platform vendors provide quality scores for individual bases in the sequence reads. These quality scores are utilized by TileQC[1] and PIQA[2], as well as vendor provided solutions for plotting quality score trend in sequencing cycles and quality score distribution in each tile, which is the unit of image capture for the Illumina platform. TileQC also plots genomic alignment results based on number of mismatches on individual tiles and such graphs are very helpful for understanding the impact of quality issues on sequence alignment.

**Figure 1:**
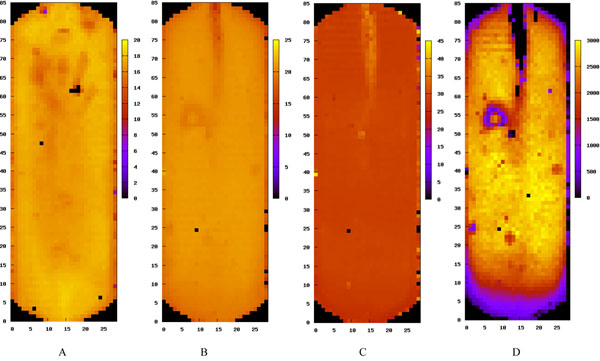
**Sample overview of full slide SOLiD runs**. Figures 1A and 1B show panel average quality score distributions across two different full SOLiD slide runs. Black areas in four corners are regions not used by sequencing assay by design. Heat map scales are set up automatically according to the quality score range in each run. The pattern of low quality regions varies from run to run even in data from the same sequencing core. Figures 1C and 1D show the color code 0 percentage distribution and the genomic hit count distribution across different panels for the same sample illustrated in Fig. 1B, respectively. Numbers on the left and lower edge of each figure are the row and column number of panels. Numbers of the right side of the heat map scale bar are values associated with colors in the heatmap.

**Figure 2:**
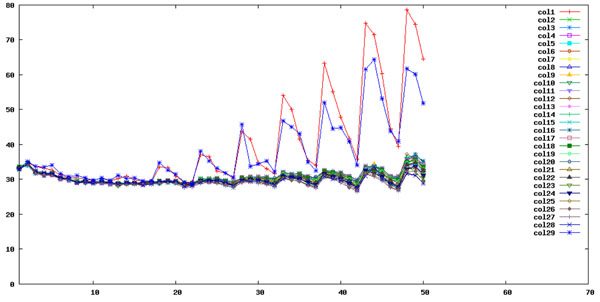
**SOLiD color code bias during the sequencing by ligation process.** Each line in Figure 2 represents the fluctuations of SOLiD color code zero for each one of the 29 columns of panels obtained in Figure 1B during the SOLiD sequencing process. The y-axis is the percentage of color code 0 at different sequencing cycles shown on the x-axis.

Besides incorporating key QC measures from existing solutions, we add the following novel QC measures in NGSQC for detecting quality issues that other solutions cannot identify:

1. Base/color code distribution across each tile/panel. In the sequencing data that we have processed, we have noticed that the percentage of a specific base (e.g., percentage of A bases in all called bases in Illumina sequencing or percentage of color code 0 in Applied Biosystem sequencing) in a fixed size area often exhibits non-random patterns across the sequencing surface (e.g., Figures 1C). Although the base composition (or the dibase composition in the case of SOLiD sequencing) is unique for each sample, we would expect that the distribution of sequence fragments on the sequencing surfaces be random. As a result, the distribution of base/color code should be fairly consistent across the sequencing surface if the surface area used for deriving the base percentage includes hundreds or even only dozens of sequences. Any deviation from the expected random distribution of sequences, e.g., the uneven concentration of A base rich sequences in a sub-region of the sequencing surface, would suggest problems in sequencing assays.

2. Base/color code percentage plot in full sequencing cycle for all sequences in each tile/panel. Similarly, we noticed cyclic patterns for the percentage of specific base/color code call along the full sequencing process, in particular in the later cycles of the sequencing assay for some samples (Figure 2). Since the widely-used quality score plots cannot predict base/color code bias, the base/color code percentage trend plot can reveal new sources of sequencing problems not detectable by the quality score plots.

3. Summary of all tile/panels based on x-y coordinates in each tile/panel: In our analysis of deep sequencing data, we also noticed that summarizing a specific quality measure (e.g., quality score or base percentage) for the same xy location for all tiles/panels in a sample can sometimes reveal patterns not obvious when these measures are examined at individual tile/panel levels. Conceivably, the summary across all tiles/panels will make weak but repetitive or consistent biases that the sequencing system introduced on each tile/panel more obvious. A common problem is likely to be the inappropriate imaging capture system setup, as donut-like patterns or stripes can sometimes be seen on the data we examined.

4. Full sample view: Since the individual tile/panel based visualization cannot capture large scale quality pattern across the full sample assay, NGSQC provides full sample view of several quality control measures derived from individual tiles. There are two different views for all tiles/panels in a sample: 1) heat maps for all tile/panels based on their original spatial relationships on the sequencing chip/slide. 2) cycle-based plot for QC measures derived from all tiles/panels in individual rows or columns in a sample. These graphs allow users to quickly identify the pattern of problematic tiles/panels, and thus can use tile/panel location as a filter in sequencing analysis.

5. Quality measures for paired-end/mate pair libraries: Although sequencing library preparation is not part of the deep sequencing assay *per se*, paired end/mate pair sequencing library preparation procedures have major influences on the final sequencing results. We included two quality measures, the distribution of distance between sequencing reads from the two ends and percentage of chimeras, in the NGSQC package for paired-end/mate pair libraries.

### Identifying potential quality issues in sequence reads related to specific biological conclusions

An important reason that quality control analysis is not widely used by biomedical researchers is that although existing solutions can reveal a number of quality issues, it is hard to investigate the biological consequences of these quality issues. A key feature provided by NGSQC is the ability to link sequence reads related to a specific biological conclusion (e.g., a list of differentially expressed genes, novel alternative splicing variants, etc.) to potential quality issues. NGSQC will plot the user-provided sequence lists back to the original sequencing surface as sequence distribution heat maps, which can help identifying potential quality issues related to specific biological results.

The most straightforward method is to examine the sequence read distribution in the sample/tile maps. Since we expect sequences related to a specific biological conclusion be distributed relatively evenly across the sequencing assay surface, any significant deviation from random distribution, either at the full sample overview or at the all tile/panel summary view, will suggest the involvement of sequencing quality issues. The expected random distribution of sequencing reads is a surprisingly powerful property that has not previously been utilized in quality control solutions. We believe that the deviation of random distribution can detect many types of quality issues even if we do not understand the underlying mechanisms.

In addition, our solution provides side-by-side comparison of the user-defined sequence reads distribution with different types of quality control heat maps generated by NGSQC for the full sequencing assay. The side-by-side comparison allows users to quickly identify situations where most of the sequence reads related to a specific biological conclusion are from problematic regions revealed by the heat map of a specific quality measure through visual pattern match. This approach is particularly useful for identifying quality issues in a small number of sequence reads related to rare genome structure changes or transcriptome variants, as it is hard to judge the randomness of distribution for a small number of sequence reads.

### Multiple deep sequencing platform support

Currently, neither TileQC nor PIQA supports sequencing data from the Applied Biosystems SOLiD platform, which is gaining popularity for SNP identification and genomic structure analysis. It is highly likely new deep sequencing platforms will become available in the coming years. NGSQC is designed to support all sequencing technologies that perform sequencing on a two dimensional surface, as long as they provide methods for linking individual sequence reads to specific locations on the sequencing surface.

In NGSQC, we use the sequencing format files to capture the two-dimensional arrangement of different sequencing assay types for a sample. For each new sequencing format from an existing or new sequencing platform, we will add a new sequencing format file that defines the arrangement of tiles/panels in each sample as well as the number of x and y values in each tile/panel.

For each analysis, NGSQC will ask a user to select a sequencing format file out of a list of all supported platforms. NGSQC will find corresponding sequencing format file under the conf folder, which is created during NGSQC pipeline setup, for generating the heat maps as well as tile/panel row/column based statistics. For example, if the user selects 'Illumina_120', the pipeline draws the image with file 'conf/Illumina_120.tile', which is an ASCII file for the one lane per sample sequencing on the current generation of Ilumina Genome Analyzer.

Since the sequencing format files are ASCII files, users of the NGSQC package can follow the example of existing sequencing format files for new sequencing assay formats or new platforms. However, if a new sequencing platform is very different, we will be happy to work with users and the corresponding vendor to generate new sequencing format files.

### NGSQC software architecture

NGSQC performs various analysis and reporting tasks in a pipeline. Its workflow is controlled by a GNU Make file, which calls a number of programs for sequence alignment (BOWTIE [9]), quality graph generation (gnuplot [10]), as well as custom LINUX scripts and Python programs for processing various text files.

Users of NGSQC can take advantage easily of multi-processor/core computers by specifying a number of jobs that can run simultaneously. NGSQC can be run on LINUX clusters too. Both Torque and SUN/Oracle Grid Engine are supported.

### NGSQC usage

To set up the NGSQC pipeline, a user needs to download a ngsqc_version.tar.gz file at http://brainarray.mbni.med.umich.edu/brainarray/ngsqc/. After unzipping it, he/she needs to go to the ngsqc folder and 1) copy genome (or transcriptome) fasta file to INPUT/fasta. 2) if the sequence reads are from the Illumina platform, copy the fastq file to INPUT/solexa. If the sequence reads are from the Applied Biosystems platform, copy both the csfasta file and qual file to INPUT/solid.

If a user wants to analyze a different type of target sequence or perform quality analysis for another species, he/she needs to perform the above procedures at another location and copy the required files.

For paired-end analysis, a user needs to create a file '<PAIRID>.pair' under either sub-folder INPUT/solid or INPUT/solexa. This file has two lines: the first line 'pair: <END1> <END2>' defines the two sequence read file names separated by a white space. These two sequence files contain sequence reads from two ends of the same sequencing library. Naturally, sequence read files END1 and END2 must exist under INPUT/solexa or INPUT/solid. The second line is 'range: 500 5000', which defines expected minimum and maximum distance between the sequence reads from two ends. In the above example, NGSQC will consider any paired-end reads with distance within 500 bp or over 5000 bp as abnormal. The NGSQC will report the number of such paired-end reads as quality measures for paired-end/mate pair library preparation.

To run the NGSQC, a user only needs to type 'make'. Users can also tweak some run parameters in the 'Environment Section' of the makefile. For example, if NGSQC is run on a big memory computer, a user might want to specify a bigger memory for program 'sort' to get better performance.

To view the result, a user can directly access file 'index.html' under the folder OUTPUT. He/she can also run 'make httpserver', which will start a web server using 8080 as the default port. The default port can be specified in makefile too.

If a user wants to perform QC analysis for several lists of sequence reads related to specific biological targets or conclusions, he/she can create sub-folders under INPUT/solexa or INPUT/solid, with one sub-folder for each sequence list. The subfolder name should be the same as the name of the corresponding sequence read file but without a suffix. Each line of such a custom sequence read file should be the sequence read ID of the corresponding sequence. After copying the custom sequence read files to their destination folders, the user only needs to run ‘make’ again to generate read distribution maps for a side-by-side comparison of the full sample QC heat maps described in earlier sections.

To utilize multi-core/CPU computer, a user can run 'make -j N', where N is a number, to run N analyses simultaneously. To run the pipeline on cluster, a user needs to modify the 'CLUSTER' parameter to either 'SGE' or 'TORQUE' in Makefile, and run 'make'. All analyses will be split into multiple smaller units and then be submitted to the cluster. The NGSQC pipeline will ensure smaller analysis units with dependency relationships executed in proper order.

Detailed usage is described in the 'README' file. The NGSQC package comes with some sample files and a user can just run 'make' for a test after unzipping. The NGSQC project website at http://brainarray.mbni.med.umich.edu/brainarray/ngsqc/ will also provide updated usage information, sample data files and sample output.

### NGSQC output

NGSQC generates an html file that organizes the quality measures in a three-layered hierarchical output. At the first level, quality control outputs are listed by lane/sample names used in the input data file. Once a sample/lane name is clicked, it will open a window displaying the quality control measures associated with this sample. Clicking on individual quality measures will show the corresponding quality analysis results in heat maps, line graphs or bar charts. The “<help>” signs on the right side of each QC measure are linked to examples on the NGSQC project webpage, if more detailed explanations are needed. The following is a brief summary of the output.

1. Full Sample View: provide sample level overview of several QC measures, including the distribution base/color code, target hit count under different mismatch criteria and target hit levels (unique or multiple), sequencing read density, and quality score based on the corresponding average values for each tile/panel used by a sample. The results are presented in the same spatial layout as it is the deep sequencing assay to facilitate a quick identification of trends/patterns of quality issues in the whole sample assay.

2. All Tiles/Panels Summary View: The above quality measures from all tiles/panels are used to create heatmaps based on individual x-y locations on the two dimensional tile/panel surfaces. This set of QC graphs is designed for detecting QC problems that are repeated for every tile/panel.

3. Individual tile/panel QC: Individual tile QC maps can be used for identifying QC issues in individual tiles. To facilitate quick identification of problematic tiles/panels, we try to rank the unevenness of two measures, the read count and the genomic hit on each tile/panel, across x-y coordinates. Currently, we use a simple fixed grid for detecting unevenness.

4. Cycle-based QC plot: the average of quality measures from all tiles/panels as well as rows and columns of tiles/panels plotted against the base/color position in the sequence reads. We also include the mismatch rate derived from unique genome hits for each base position along the full-length sequence reads. These graphs will not only help to identify cycle-specific sequencing issues but also spatial-related issues based on tile/panel rows and columns.

5. Target hit plot: These graphs present sequence alignment results across the target genome or transcriptome sequences. They are useful for identifying uneven distribution of sequences when compared to reference targets or help to identify sequence structural differences between the sample and the reference genome/transcriptome.

6. QC for user-defined sequence lists. If a user analyzes the lists of sequences related to specific biological conclusions, the resulting QC data will be listed under the above categories in the output. The QC graphs for user-defined sequences will be displayed side-by-side with the QC graph for all sequences in the sample thus users can quickly find out whether their interested sequences are from problematic regions of sequencing assay.

7. Library QC for paired-end/mate-pair sequencing: The paired-end/mate-pair library overview graph presents the percentage of good pairs (correct orientation on the same chromosome), unpaired reads, chimeric pairs from different chromosomes, chimeric pairs with the wrong orientation from the same chromosome, and chimeric pairs less than or greater than the user-defined library fragment range in a bar chart. The pair distance distribution plot can be used to judge whether the matched paired-end/mate-pair reads exhibit the correct distance distribution. The distance between each good pair is calculated by the starting position of the first end, minus the starting position of the second end, thus the distance values can be negative. We also plot pairs hitting different strands of the target separately. As a result, the pair distance distribution plot can also be used to detect strand bias.

## Results and discussions

The NGSQC program we developed has several important advantages over existing Illumina sequencing quality control solutions such as TileQC and PIQA: 1) NGSQC incorporates the most comprehensive quality control measures, including the novel base bias detection on sequencing surfaces and in sequencing cycles, full sample view for identifying large scale trend/unevenness, all tiles/panels summary view for detecting repetitive optical and fluidic problems and QC graphs for assessing paired-end/mate-pair library quality issues. 2) NGSQC enables easy identification of quality issues related to specific biological targets/conclusions by plotting user-defined sequences in the same QC pipeline. 3) NGSQC is the first quality control package for the SOLiD platform and it can be easily adapted for any future sequencing technologies as long as the sequencing is performed on a two dimensional surface. 4) NGSQC supports cluster computing, thus it is suitable for large sequencing projects.

The application of the NGSQC pipeline to various deep sequencing data sets revealed unexpected quality issues, which helped us to derive more reliable biological conclusions. While detailed discussion of the related biological issues is beyond the scope of this paper, we would like to use three examples to demonstrate how NGSQC can be utilized to identify potential quality problems and to improve deep sequencing data interpretation.

Example 1: Quality issues in a full SOLiD slide run:

We start with the SOLiD example since there was no QC analysis package for the SOLiD platform before NGSQC. Figures 1A and 1B show the sample level overview of the individual panel quality score average in two different full SOLiD slide runs from two different sequencing labs. It is obvious that sequence data from different labs produce different quality score distributions. In fact, the quality score distribution varies from run-to-run even in the same lab. The region of low quality scores near the upper middle part of the SOLiD slide in Fig. 1B actually has a good explanation: samples are injected into the slide through an opening at the top of the slide, thus the band of low quality region is likely caused by fluidics issues.

Figures 1C and 1D show the distribution of the color code 0 percentage and the genomic hit count across different panels for the same sample as in Fig. 1B. Both the color code bias and the genome hit count across the panels reflect at some degree the SOLiD quality score distribution near the upper middle part as described above. However, the magnitude of quality difference across different panels is greatly magnified in the genome hit plot, suggesting that the final genome hit count is a more sensitive measure for detecting quality issues in sequencing assays.

Figure 2 shows the color code bias in the SOLiD sequencing by ligation process: This is the average color code bias for each column of panels (for a total of 29 columns) of the sample used in Figures 1B, 1C and 1D across dinucleotide extension cycles. It is surprising to see the SOLiD sequencing by ligation process, which consists of 5 rounds of primer reset for each sequencing tag (http://www.appliedbiosystems.com/absite/us/en/home/applications-technologies/solid-next-generation-sequencing/next-generation-systems/solid-sequencing-chemistry.html ), also lead to cyclic dinucleotide bias (every 5 dinucleotide extensions) in parallel to the primer reset cycles. Obviously, the color code bias problem is most severe near the end of sequencing.

In addition, it can be seen that column 1 and column 29 exhibit very different color code bias changes in the sequencing process. This corresponds well to other quality measures in Fig. 1B, 1C and 1D (darker regions in the leftmost and rightmost columns). Consequently, it will be better to exclude sequence reads from column 1 and column 29 in the final analysis for this particular run.

**Figure 3:**
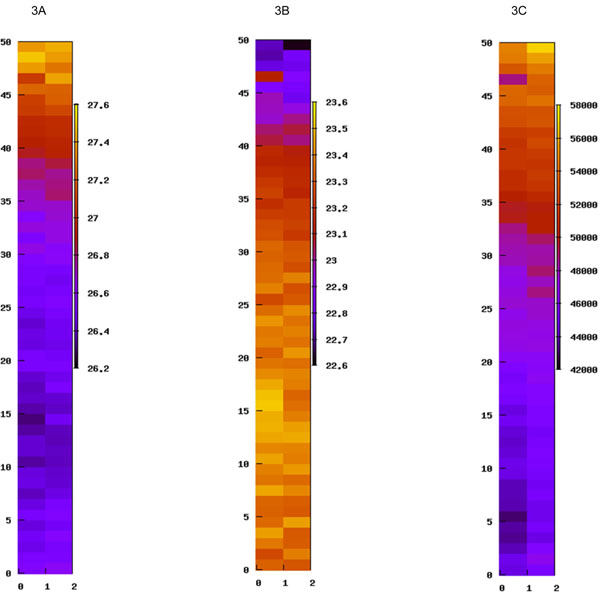
**Small but consistent spatial base percentage gradient in Illumina sequencing.** Figures 3A, 3B and 3C show the average percentage of the A base, average percentage of the C base and the genome hit count in each tile within an Illumina lane for the same sample. The x-axis labels and the y-axis labels on the left side are the column and row numbers of tiles. The y-axis labels on the right side of the heatmap scale are the values associated with heatmap colors.

Example 2: Small but consistent base bias across Illumina lanes:

Figure 3A and 3B show the A base percentage and the C base percentage for each tile within a lane on the Illumina sequence chip. There is a small (1%-2%) but consistent base percent different at two ends of each lane. Figure 3C is a heat map based on the number of sequences with unique genome hit count per tile (from 42000 to 58000). Again, genomic hit count is a more sensitive quality measure than base percentage change or the vendor provided quality score.

Nonetheless, base percent change heat map can help users to better relate their sequence data to biological problems. For example, if a user is looking for a rare structural change involving sequences with high C base content, he/she should become alarmed if the NGSQC user sequence mapping function reveals that most of those sequences are from the end of the lane containing higher level of C bases. It is likely that sequencing assays artificially introduced more calls for C bases in the sequence reads to enable the alignment of those sequences to the expected target, such as new exon-exon junction or a genomic insertion/deletion. Such candidates should definitely be given lower priority in follow-up validation experiments.

We would like to point out that base bias and sample level unevenness in sequencing quality are very common issues in deep sequencing. However, neither TileQC nor PIQA is able to detect the related quality issues directly since they neither use base bias as a quality control measure nor provide full sample view for detecting large scale trends.

**Figure 4:**
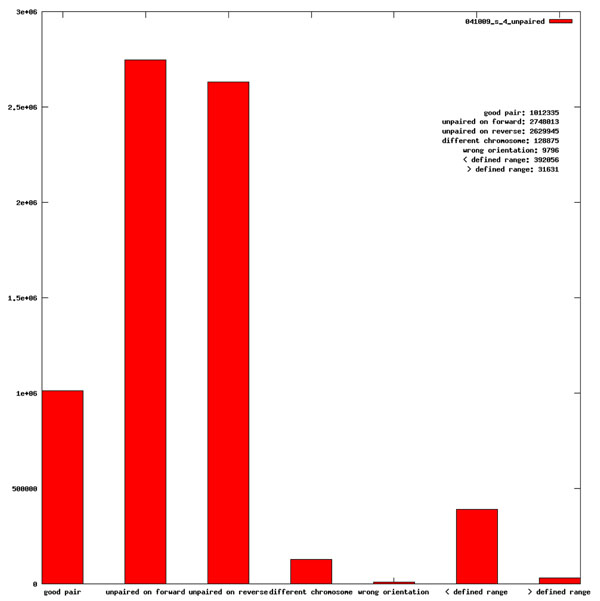
**Quality control for paired-end sequencing.** The y-axis shows the number of pairs for each of the following categories: 1) good pair: sequence reads from both ends of a sequence are from the same chromosome and their distance and orientation are consistent with the reference genome; 2) unpaired on the forward strand: orphan reads from one end of the sequencing; unpaired on the reverse strand: orphan reads from the other end of sequencing. We separate the reads from two ends since for some technologies the reading efficiency and accuracy are different for two ends; 3) different chromosome: two ends of the same fragment are from different chromosomes based on the reference genome; 4) wrong orientation: although the two ends are from the same chromosome, their relative orientation is different from the reference genome; 5) < defined range: paired-end reads with shorter than the expected library fragment range and 6) > defined range: paired-end reads with longer than the expected library fragment range. In the above example, more than one third of the pairs have a shorter than expected distances, thus indicating a library quality issue.

Example 3: Paired-end/mate-pair sequencing quality measures:

Paired-end or mate-pair sequencing are widely used in analyzing larger scale genome or transcriptome structural changes such as large insertion, gene fusion and alternative splicing. A key issue in paired-end/mate pair data analysis is the false chimeric sequences, whose two ends are not from the original continuous sequence fragments during library preparation. NGSQC can generate a barchart summarizing the extent of chimeric sequences in the results based on unique genome hits (Figure 4). Consequently, users can quickly learn the fraction of two ends from different chromosomes, and two ends with wrong orientations, as well as those with longer than or shorter than expected distances. Such chimeric sequences are in fact candidates for gene fusion, inversion, insertion and/or deletion. However, if their levels are very high, it is likely that many such “novel” genomic structural changes are caused by problems in library preparation. For example, Figure 4 shows the QC results for a public domain paired-end sequencing sample from the 1000 genome project [11,12]. About 10% of the paired end reads in that sample have ends from different chromosomes according to the current version of genome assembly (hg19). Consequently, most of the genomic structural changes revealed by paired-end sequencing for this subject must be the result of poor sequencing library preparation, as it is highly unlikely that the corresponding subject can survive with such a high level of genomic disruptions.

## Conclusions

NGSQC is a comprehensive deep sequencing quality control pipeline that can help biomedical researchers quickly find out if there are specific quality issues related to their results. We expect that the incorporation of NGSQC in standard deep sequencing data analysis pipeline can significantly improve the interpretation and understanding of deep sequencing data. We also welcome feedback from the deep sequencing community for further improvement of NGSQC.

## Availability and requirements

• **Project name:** NGSQC

• **Project home page:**http://brainarray.mbni.med.umich.edu/brainarray/ngsqc/

• **Operating system(s):** LINUX

• **Programming language:** gnu make, shell script and python

• **Other requirements:** BOWTIE, gnuplot and Sun Grid Engine or TORQUE as cluster manager if running NGSQC on a LINUX cluster

• **License:** GNU GPL

• **Any restrictions to use by non-academics:** License needed

## Authors’ contributions

MD designed the software architecture, implemented NGSQC, performed the majority of data analysis and helped to draft the manuscript. RCT, CM and RC carried out most of the deep sequencing studies. RCT, CM, RC, MHK, DMM and GO helped the interpretation of biological significance of quality control measures, provided critical feedback for NGSQC improvements and revised the manuscript. FM conceived the project, designed the main NGSQC functions, participated in data analysis and drafted most of the manuscript.

## Competing interests

The author(s) declare that they have no competing interests.
